# Extreme drought and sexual violence against adolescent girls and young women: A multi-country population-based study

**DOI:** 10.1371/journal.pgph.0004752

**Published:** 2025-06-26

**Authors:** Lucas Hertzog, Marshall Makate, David Chipanta, Boladé Banougnin, Martina Mchenga, Gavin Pereira, Sylvester Dodzi Nyadanu, Jennifer Dunne, Paula S. Tallman, Shalean Collins, Kefyalew Addis Alene, Pauline Rousseau-Gueutin, Astghik Mavisakalyan, Ivan C. Hanigan

**Affiliations:** 1 Curtin School of Population Health, Faculty of Health Sciences, Curtin University, Perth, Australia; 2 WHO Collaborating Centre for Climate Change and Health Impact Assessment, Perth, Australia; 3 Healthy Environments and Lives (HEAL) National Research Network, Canberra, Australia; 4 United Nations Joint Programme on HIV/AIDS (UNAIDS), Windhoek, Namibia; 5 United Nations Population Fund, West and Central Africa Region Office, Dakar, Senegal; 6 Centre for Social Science Research, University of Cape Town, Cape Town, South Africa; 7 enAble Institute, Faculty of Health Sciences, Curtin University, Perth, Australia; 8 Department of Anthropology, Loyola University Chicago, Chicago, Illinois, United States of America; 9 The Keller Science Action Center, The Field Museum of Natural History, Chicago, Illinois, United States of America; 10 Department of Social Welfare, Faculty of Social and Political Sciences, Padjadjaran University, Bandung, Indonesia; 11 Department of International Health & Sustainable Development, School of Public Health and Tropical Medicine, Tulane University, New Orleans, Louisiana, United States of America; 12 Geospatial and Tuberculosis Research Team, Telethon Kids Institute, Perth, Australia; 13 Department of Global Health and Social Medicine, Harvard Medical School, Boston, Massachusetts, United States of America; 14 Univ Rennes, Ecole des Hautes Etudes en Santé Publique, Rennes, France; 15 Bankwest Curtin Economics Centre, Curtin University, Perth, Australia; 16 ARC Centre of Excellence for the Elimination of Violence Against Women, Melbourne, Australia; 17 Centre for Safe Air, NHMRC CRE, Hobart, Australia; ENS: Ecole Normale Superieure, FRANCE

## Abstract

Droughts have profound and far-reaching impacts on human health and well-being, but their influence on sexual violence among adolescent girls in low- and middle-income countries (LMICs) is underexamined.This study examines the association between drought and sexual violence against adolescent girls and young women globally, using cross-sectional, nationally representative data from the Violence Against Children and Youth Surveys (VACS) from 2013 and 2019. The sample includes 35,309 females aged 13–24 from 14 countries in Central and South America, sub-Saharan Africa, Southeast Asia, and Eastern Europe. Sexual violence was defined based on unwanted sexual contact, completed or attempted forced penetration, and pressured sexual activity within the past 12 months. Drought exposure captured intensity and duration of drought conditions measured using the Global Standardized Precipitation-Evapotranspiration Index (SPEI) over a 48-month period before surveys. Bayesian Generalized Linear Models were employed to estimate the association between drought exposure and reported experiences of sexual violence, controlling for age, relationship status, school attendance, and wealth. The analysis revealed that exposure to prolonged and extreme drought, lasting 8–43 months in a 48-month period, was associated with higher odds of sexual violence (OR 1.21, 95% CI 1.21 to 1.22). Very dry periods are also associated with increased odds of experiencing sexual violence (OR 1.04, 95% CI 1.04 to 1.05). In contrast with extreme conditions, exposure to slight to moderate drought and recent and long periods suggested potential protective effects.This study provides novel evidence of an association between extreme drought and an increased likelihood of sexual violence against adolescent girls and young women in LMICs. The findings emphasise how climate change can exacerbate social vulnerabilities through its indirect effects, highlighting the need for comprehensive assessments of its impact on vulnerable populations.

## Author summary

**Why was this study done?:**

- Extreme weather events, such as droughts, are known to have wide-ranging consequences on human health, and their links with sexual violence against adolescent girls and young women have not been quantitatively studied.
- Previous research has linked drought to risk factors for gender-based violence, including poverty, migration, and water insecurity. Still, no population-level analysis has focused specifically on sexual violence in younger women and adolescents.

**What did the researchers do and find?:**

- We combined nationally representative survey data from 14 low- and middle-income countries with detailed drought exposure data, estimating the association between drought and sexual violence among 35,309 adolescent girls and young women aged 13–24 years.
- Extreme drought conditions were associated with a significantly higher risk of sexual violence, while non-extreme drought conditions may potentially act as protective factors.

**What do these findings mean?:**

- Exposure to extreme drought conditions may exacerbate risks of sexual violence against adolescent girls and young women, and gender-sensitive climate change adaptation policies are urgent.
- Future research should explore the mechanisms linking environmental stressors across time to increased risks of sexual violence to inform targeted interventions in vulnerable regions.

## 1. Introduction

Extreme weather events such as droughts have profound and far-reaching impacts on human health and well-being [1–3]. These events are becoming more frequent and intense due to accelerated unsustainable anthropogenic activity, with significant implications for lower and middle-income countries at higher risk of current and worsening environmental exposures and lack of system preparedness for climate change-associated events [4,5]. Droughts have been linked to reduced agricultural productivity, food and water insecurity, and economic hardship [6], and various adverse health outcomes such as respiratory diseases and mortality [7–9], cardiovascular mortality [10,11], mental health disorders and suicide [12–14]. However, one of the less explored yet critical impacts of drought is its potential to exacerbate sexual violence, specifically against girls and young women.

Multiple pathways, on the individual, household, and community levels, may underlie the association between drought and sexual violence. For example, on the individual level, drought conditions can increase the likelihood of engaging in transactional or survival sex, which subsequently increases the risk of forced sex, other forms of sexual violence, and HIV acquisition [15]. At the household level, droughts are associated with increased practices of female genital mutilation and early and/or forced marriage as families attempt to off-load family members (particularly girls) as they navigate increasing resource scarcities [16–20]. Finally, on the community level, droughts can produce water insecurity, which is associated with opportunistic violence that occurs when women and girls are collecting water and include kidnapping, bridal abductions, sexual harassment, assault, and rape [21].

While there are multiple mechanisms linking droughts with sexual violence, whether these associations hold across different global contexts remains unknown. This study aims to fill these gaps by investigating the association between exposure to drought and the likelihood of sexual violence against girls and young women across multiple low- and middle-income countries (LMICs) using large-scale, nationally representative surveys. By quantifying the impact of different drought conditions on the odds of experiencing sexual violence and controlling for sociodemographic variables, this analysis provides an empirically based example of how climate change and related environmental stressors can heighten risks for already vulnerable populations.

## 2. Methods

### 2.1. Ethics statement

Following WHO Guidelines, consent was obtained from parents or legal guardians when participants were under 18 years old, and informed assent/consent was obtained from all participants. Relying on best practices in interviewing individuals under 18 and focusing on safeguarding participants from potential negative consequences in responding to the survey, parents and guardians were informed that questions to their children would be sensitive. Still, they were not informed of the entire nature of the survey [22]. Participants were interviewed inside or outside their residences, where no one else could hear them, with the approval of their caregivers. They were informed that they had the right to withdraw from the survey at that time. Trained data collectors administered the survey, and participants’ confidentiality was guaranteed. Survey data were de-identified and securely curated to safeguard the participants’ privacy, adhering to best practices in data management with sensitive data [23]. Research and Ethics Committees reviewed and approved survey protocols across partner governments in countries and by the CDC and Columbia University Institutional Review Boards.

### 2.2. Study design and sampling

The Violence Against Children Surveys (VACS) are nationally representative cross-sectional household surveys conducted to assess the prevalence of violence experiences among children and young people across 23 countries. From these, 14 were eligible for this study (survey data collection year in parenthesis): Cambodia (2013), Colombia (2018), Côte d’Ivoire (2018), El Salvador (2017), Kenya (2018–19), Lesotho (2018), Malawi (2013), Moldova (2018), Mozambique (2019), Namibia (2019), Nigeria (2014), Uganda (2015), Zambia (2014) and Zimbabwe (2017). Eight countries with available data were excluded for diverse reasons (see sampling details in S1 Text). All included countries had comparable surveys and information on participants’ residence at the district level (except Mozambique, which only had provinces).

### 2.3. Outcome: Sexual violence

The outcome variable was measured using a combination of questions (Table A in S1 Text) that aimed to capture various forms of sexual violence victimisation involving a lack of freely given consent and situations in which the victim is unable to consent or refuse [24]. Questions were asked to the selected 13–24-year-old female respondents and focused on the following types of sexual violence: 1) unwanted sexual contact (touching sexually without permission including fondling, pinching, grabbing, or touching on or around sexual body parts), 2) completed or attempted forced penetration (vaginal, oral or anal sex or the insertion of hands, fingers or other objects in the victim’s vagina or anus by someone else), 3) non-physically forced penetration after the victim is pressured verbally or through intimidation or misuse of authority (pressured into sex). Multiple questions were posed to participants about lifetime occurrence, with follow-up questions about the perpetrator, circumstances, and whether the event happened in the 12 months preceding the interview. Recent sexual violence (previous 12 months) was selected as the dependent variable and operationalised as disclosure of any of the above questions and an affirmative response to the follow-up question if the violence had occurred in the prior year.

### 2.4. Drought measures and sensitivities to change in exposure

Dryness patterns, particularly drought conditions at the extreme end, in each participant’s region were identified using standardised precipitation and evapotranspiration index (SPEI) calculated on a 0.5º grid (approximately 50km × 50km). SPEI is a multiscalar index that enables the identification of various drought types, and it is suited for studies that aim to capture the effects of climate change on drought severity [25,26]. It is a linear scale ranging from -4 (dry) to +4 (wet) that can be calculated at different time scales. This study used a 6-month SPEI to estimate drought exposure categories, a scale previously used to capture drought impacts on mental health outcomes [27].

Using SPEI, we estimated two drought indices to represent intensity and/or duration, aligned with the time window of the interviews. The method used to estimate those drought indices is presented in detail in S1 Text. A brief presentation of the different steps is summarised below.

Months with SPEI values below or equal to -1 were considered dry months. Considering those dry months, the count and the sum methods were estimated. The count method determines the number of dry months characterising a drought, which starts in a period of at least five consecutive dry months. The count method thus captures the duration of the drought. To capture the intensity of the drought, we used the sum method, which sums the SPEI values of consecutive dry months. When the sum of the SPEI values was less or equal to -17.5, then a drought was defined considering the intensity [28]. A total of drought months that met both conditions (duration via count method and intensity via sum method) was estimated. Finally, we calculated the number of drought cycles using each method (count and sum) and the cycles in which both were met. Each drought cycle represents an independent period of drought, starting with the first month of dryness that satisfies the criteria for either duration or intensity and ending when conditions no longer meet these thresholds, allowing us to identify and quantify distinct drought events over time.

A third method estimated a standardised dryness intensity to compare areas with extreme conditions and create a category of intensity only (Table F in S1 Text). It was calculated as the sum during the periods below the threshold (-17.5) divided by the average time per drought cycle (total number of drought months divided by the number of cycles in the sum method).

These methods were used to characterise four drought categories created to capture drought intensity and duration/recency and similar methods were previously used in a study to estimate the indirect impacts of environmental exposures associated with drought [27] with similar methodologies used elsewhere [14,29].

Two categories characterise intensity. The first category, slight to moderate, corresponds to slight to moderate dryness levels (monthly SPEI of less than zero to -1). The second, very dry, corresponds to intense dry periods (top 10% of the months across the sample using the standardised dryness intensity).

One category focuses on duration only: recent and long (more than 12 consecutive dry months in the 24 months before survey data collection). Finally, prolonged and extreme (top 10% of months in a drought that met the sum and count methods definition), characterises duration and intensity, focusing on the most extreme periods.

The drought exposure measures were calculated for grid cells and averaged for the small-area geographical level of 13 countries using information from their census districts (administrative area level two). For Mozambique, district-level data was unavailable, and provinces from the country’s census were used. The denomination of administrative area levels was chosen to link the drought measures with the population data taken from the VACS. Drought data used for calculating the exposure experienced by individual respondents was selected to match a 48-month time period before interviews.

### 2.5. Covariates

The model controlled for socio-demographic factors that are well-documented in the literature as associated with both sexual violence and environmental stressors such as drought: age (continuous, in completed years), school attendance (binary, participants currently attending school), relationship status (binary, ever married or in a romantic relationship), and wealth (composite measure using questions associated with sanitation, assets ownership and dwelling characteristics – see Table B in S1 Text) [30–32]. Wealth was operationalised as a binary variable derived from country-specific wealth quintiles, categorising individuals in the lowest quintile (quintiles 1 and 2, representing the most economically disadvantaged) and those in quintiles 3–5. This measure reflects relative wealth within each country and accounts for socio-economic disparities rather than absolute poverty across countries.

### 2.6. Statistical analysis

We used Bayesian modelling of the survey after initial descriptive analyses were conducted. The associations between exposure and outcome were examined using a random effects Bayesian Generalised Linear Model with varying intercepts to account for unobserved heterogeneity across countries, controlling for sociodemographic characteristics. Bayesian analysis was chosen for this study due to its ability to provide direct probabilistic interpretations of model parameters, handle complex hierarchical structures more effectively than frequentist methods and facilitate the interpretation of statistical conclusions [33].

The likelihood function for the model is a binomial logistic regression, where the probability of the dependent variable (sexual violence) being 1 (i.e., the event occurring) is modelled as a function of the predictor variables and control variables (see formal specification in S1 Text). Priors for the model parameters were chosen to be weakly informative, centred around zero, with a standard deviation of 1. This reflects a belief that a priori, there is no strong expectation about the direction or magnitude of the effects but allows the data to inform the posterior distributions. Posterior predictive checks were conducted to ensure the model adequately captured the data. Density overlay plots were generated to compare the observed data with the posterior predictive distribution, demonstrating that the model can replicate the observed patterns. The central tendency of the posterior distributions for continuous parameters was summarised using the median, with 95% credible intervals (CI) reported for each parameter. The results provided probabilistic interpretations of the impact of drought on the likelihood of sexual violence. Convergence and resolution of the Markov Chain Monte Carlo (MCMC) chains were assessed using trace plots and the potential scale reduction factor (*R̂*), and Effective Sample Sizes (ESS) were examined to ensure that the chains had sufficient resolution. Missing values were imputed using the *missForest* package.

Stata 16.1 and R 4.2.2 were used to prepare survey data and exposure data for analyses. Main modelling was performed with R 4.2.2 using the *targets* package to manage dependencies and organise a reproducible workflow. The *exactextractr* package handled exposure data preparation, while *rstanarm* and *survey* were used for Bayesian modelling and survey design declaration. Survey data preparation in Stata involved steps conducted for each of the 14 countries, and details are provided in S1 Text.

## 3. Results

### 3.1. Descriptive statistics

A sample of 35,309 adolescent girls and young women aged 13–24 were included in the study, with an average age of 18.2 years (SD 3.5) (Table 1). Fewer than half (45%) of participants (n = 15,743) were 13-17y, 55% (n = 19,564) were 18-24y; two participants had missing age data.

**Table 1:** Summary statistics for sociodemographic variables and sexual violence experiences among 35,349 females aged 13-24 years across 14 countries.

Characteristic	N = 35,309 n (%)/ mean (SD)
Age	18.2 (3.5)
Missing	2
Age group	
13-17	15,743/ 35,307 (45%)
18-24	19,564/ 35,307 (55%)
Missing	2
Attending school	17,487/ 35,283 (50%)
Missing	26
Ever married/relationship	22,645/ 35,149 (64%)
Missing	160
Household is impoverished	14,069/ 35,309 (40%)
Sexual violence (lifetime)	7,240/ 35,251 (21%)
Missing	58
Sexual violence (previous 12 months)	
No	3,679/ 35,254 (10%)
Yes	3,506/ 35,254 (9.9%)
Not Applicable^1^	28,069/ 35,254 (80%)
Missing	55
Sample by country	
Cambodia	1,121/ 35,309 (3.2%)
Colombia	1,366/ 35,309 (3.9%)
Côte d’Ivoire	1,200/ 35,309 (3.4%)
El Salvador	1,056/ 35,309 (3.0%)
Kenya	1,344/ 35,309 (3.8%)
Lesotho	7,101/ 35,309 (20%)
Malawi	1,029/ 35,309 (2.9%)
Moldova	1,024/ 35,309 (2.9%)
Mozambique	2,129/ 35,309 (6.0%)
Namibia	4,211/ 35,309 (12%)
Nigeria	1,766/ 35,309 (5.0%)
Uganda	3,159/ 35,309 (8.9%)
Zambia	891/ 35,309 (2.5%)
Zimbabwe	7,912/ 35,309 (22%)

^1^Not applicable due to the survey skip pattern. Question only asked for those who responded ‘yes’ for lifetime sexual violence.

Sample distribution across countries varied, with the largest number of participants in Zimbabwe (22%, n = 7,912) and Lesotho (20%, n = 7,101) and smallest sample sizes in Moldova (2.9%, n = 1,024) and Zambia (2.5%, n = 891).

Half of the participants were attending school (n = 17,487), with 26 missing responses. Regarding relationship status, 64% (n = 22,645) had ever been married or in a relationship, with 160 missing responses (0.45%). A total of 14,069 respondents lived in impoverished households. Lifetime sexual violence was reported by over a fifth of the sample (n = 7,240), with nearly half of them experiencing sexual violence in the previous 12 months of the survey (n = 3,506).

**Fig 1:**
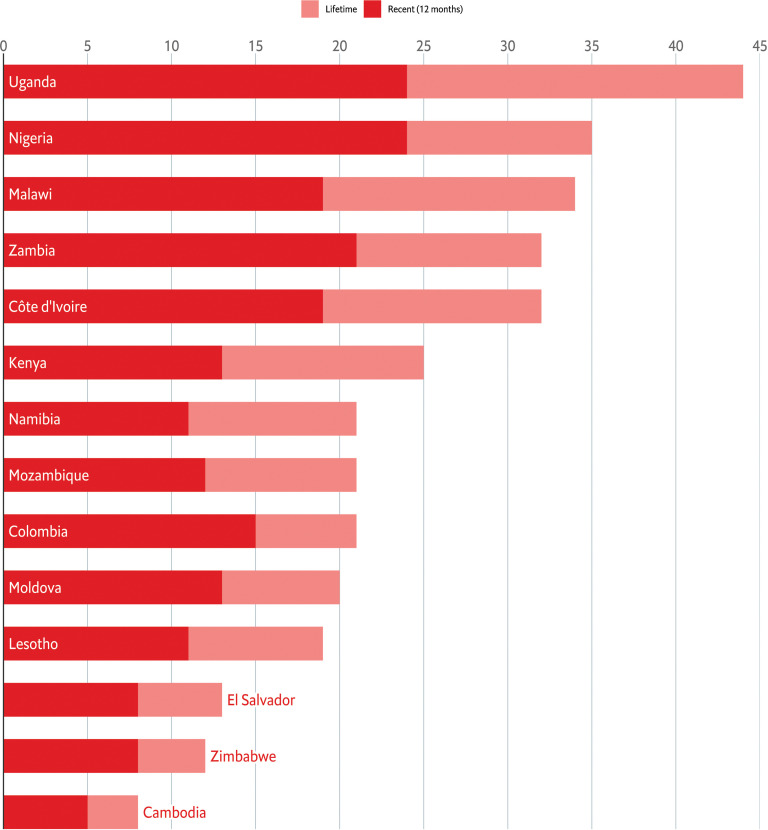
Prevalence of Lifetime and Recent Sexual Violence Among Females Aged 13-24 Years Across 14 Countries.

Fig 1 shows the prevalence of lifetime and recent sexual violence among participants across the 14 countries. Uganda had the highest prevalence of sexual violence, with 43.98% of respondents reporting lifetime victimisation and 20.69% reporting recent sexual violence. Conversely, Cambodia had the lowest prevalence, with 7.96% of respondents reporting lifetime sexual violence and 4.59% reporting recent sexual violence. Detailed estimates, including 95% confidence intervals and standard errors, are provided in Table C in S1 Text.

**Fig 2:**
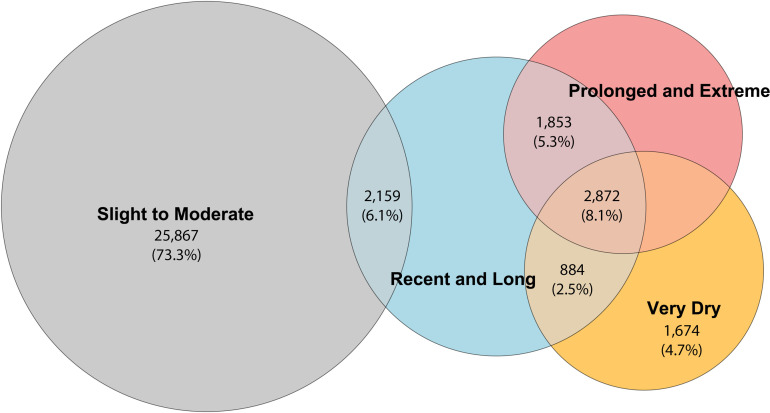
Distribution of drought conditions and sample exposure.

Most participants were exclusively exposed to “slight to moderate” drought conditions (73.3%). There was an overlap of 6.11% of participants (n = 2,159) being exposed to both “slight to moderate” and “recent and long” drought. Very dry represents intense drought periods (top 10% of the months using the standardised dryness intensity) and 1,674 participants were exposed exclusively to these dryness conditions (with an additional 884 overlapping with “recent and long”, and 2,872 overlapping with “prolonged and extreme” drought). “Recent and long” represents exposure to more than 12 consecutive dry months in the previous 24 months, and in total, 7,768 participants were exposed to these dryness conditions, with several overlaps with other categories. 4,725 participants were exposed to “prolonged and extreme” conditions (the top 10% of months in drought based on the cumulative experience that met the sum and count methods criteria). A complete distribution of exposure to dryness conditions is available in Fig 2 (for a detailed summary see Table D in S1 Text):

### 3.2. Association between drought and sexual violence

Table 2 summarises the analysis of the association between exposure to different drought conditions and the likelihood of experiencing sexual violence. The table presents the coefficient estimates, odds ratios, 95% credible intervals (CI), and posterior probabilities for each exposure category.

**Table 2:** Summary of the association between sexual violence and dryness conditions.

Exposure	Estimate	SE	OR	95% CI-	95% CI+
Slight to moderate	-0.008	0.002	0.992	0.988	0.995
Recent and long	-0.156	0.002	0.855	0.853	0.858
Very dry	0.046	0.002	1.047	1.043	1.050
Prolonged and extreme	0.197	0.002	1.218	1.213	1.222

SE: Standard error; OR: Odds ratio; CI: Credible interval.

Estimates represent the mean of the posterior distribution of the coefficient, indicating the average effect of the predictor on the log-odds of the outcome. The 95% Credible Interval (CI) represents the range within which we are 95% certain the true coefficient lies.

The OR for very dry exposure (intensity) was 1.047 (95% CI 1.043 to 1.050), and for prolonged and extreme exposure (duration and intensity) was 1.218 (95% CI 1.213 to 1.222). These exposures presented a high posterior probability (1) of the coefficient being positive.

These findings indicate that exposure to two dryness conditions (i.e., very dry, and prolonged and extreme) was associated with a significantly increased likelihood of experiencing sexual violence, as evidenced by their positive coefficient estimates, odds ratios greater than 1, 95% credible intervals that do not include 1, and high posterior probabilities. In contrast, slight to moderate and recent and long drought exposures were associated with a lower likelihood of experiencing sexual violence compared to being exposed to the other drought categories, as indicated by their odds ratios below 1, and 95% credible intervals that do not include 1. However, the magnitude of the effects for slight to moderate was negligible (OR 0.99), suggesting that while the association is statistically credible, its practical significance may be limited.

## 4. Discussion

This study broadens the literature on sexual violence and environmental exposures associated with drought by exploring the association between extreme drought and sexual violence towards adolescent girls and young women in LMICs. Using cross-sectional surveys of 35,309 adolescent girls and young women aged 13–24 from 14 countries in Central and South America, sub-Saharan Africa, Southeast Asia, and Eastern Europe from 2013 to 2019, we identified associations between exposure to extreme drought and sexual violence victimisation in pooled analyses. Adolescent girls and young women exposed to very dry drought periods, estimated using a standardised dryness intensity scale, were more likely to experience sexual violence. Participants exposed to prolonged and extreme drought, which corresponds to exposure to the worst conditions across the sample during a period of 8–43 months in a 48-month window, had an even higher risk of experiencing sexual violence. Our results indicate a small inverse association between sexual violence and exposure to slight to moderate drought conditions, and its practical significance may be limited. Exposure to recent and long drought conditions was associated with a lower likelihood of experiencing sexual violence, which may be explained by the fact that being exposed to this non-extreme drought was compared to being exposed to other drought categories.

Our findings are consistent with studies that have shown an association between drought and gender-based violence [32]. Prior studies have found links between drought, extreme weather, and rain shocks at the individual [34,35], household [16–18], and community levels [17,36,37]. Although studies have shown specific impacts of drought on child marriage and female genital cutting, to our knowledge, this is the first study that specifically interrogates the association between drought and sexual violence against girls and young women.

A recent review of water insecurity, a downstream effect of drought, and gender-based violence found that most research on the topic is conducted in LMICs, with a predominance of studies in rural settings [21]. Rural agrarian and pastoralist communities in LMICs are particularly vulnerable to environmental stressors, such as droughts [38], due to the adverse impacts on livelihoods and access to resources, which increase the risk of sexual violence by forcing reliance on distant water sources [21], necessitating migration [39], and prompting early marriage to maintain scant household resources [17–19]. Few studies have been conducted on the impacts of drought on violence in urban settings in LMICs, which is a substantial area of future research that should be explored.

In addition to standardised approaches in measuring violence, a recent qualitative study conducted in Indonesia and Peru suggests that participants categorise as a form of violence the extreme burdens associated with water insecurity, which may lead to adverse sexual and reproductive health and poor socioeconomic outcomes [31]. We argue here that violence be more broadly conceptualised to evaluate the harmful impacts that drought may have for women beyond those currently documented in the literature.

One of the main strengths of this study is that it is, to our knowledge, the first to investigate the association between drought and sexual violence against adolescent girls and young women in LMICs using a survey design specifically aimed at capturing experiences of violence with a focus on this demographic. This approach offers a unique perspective on how environmental stressors, specifically drought, can exacerbate vulnerabilities to sexual violence. Unlike many studies that focus on partnered women and intimate partner violence (IPV) [30,40], our study includes both partnered and non-partnered individuals, thus broadening the scope to understand the adverse effects of droughts on violence outside of intimate partnered relationships. Additionally, this study is the largest in terms of the number of adolescent girls who have reported experiences of sexual violence. Furthermore, our study uses a robust and validated approach to measure drought exposure, capturing the intensity and duration of drought conditions. This method acknowledges that droughts may have lagged and cumulative effects, suggesting that climate shocks can have long-lasting impacts on communities. Lastly, using a Bayesian model enhances the quality of our analysis compared to frequentist methods, offering probabilistic interpretations of model parameters and effectively handling hierarchical structures, which provides a more nuanced understanding of the data.

Despite its strengths, this study has several limitations. First, the cross-sectional design limits the ability to draw causal inferences about the association between drought exposure and sexual violence. Second, while our study covers many countries, the generalisability of the findings to other regions and contexts not included in the analysis remains uncertain. However, the nationally representative samples used and the variety of regions included provide a strong basis for understanding the associations across diverse settings. Finally, the reliance on self-reported data may introduce recall bias or underreporting of sensitive experiences such as sexual violence, potentially affecting the accuracy of our findings. Nevertheless, data collection used techniques to improve disclosure [22]. Future research should address these limitations using longitudinal designs and expanding the geographical scope of this study.

In conclusion, our study adds to the emerging body of literature on the intersection between environmental stressors and gender-based violence, specifically focusing on the impact of drought on sexual violence against adolescent girls and young women in LMICs. The findings indicate that extreme drought conditions are significantly associated with an increased likelihood of sexual violence. These results underscore the need for comprehensive strategies that address drought’s immediate environmental and economic impacts and the broader social and health consequences. As climate change intensifies, investing in research exploring these linkages and identifying effective interventions is crucial. Tailored policies and programs incorporating gender-sensitive approaches and resilience-building measures are essential to mitigate the adverse effects of climate change on vulnerable populations. By understanding and addressing the multifaceted impacts of drought, we hope to inform better protections of the health and well-being of at-risk groups and enhance the overall resilience of communities facing environmental challenges.

## Supporting information

S1 Text Table A. Sexual violence measures used in the study, including questions for lifetime experience and follow-up questions to assess temporality and frequency. Table B. Questions included in the head of household questionnaire and measures used to estimate the wealth index. Table C. Weighted percentage prevalence of sexual violence victimisation in 14 countries using VACS data, including 95% confidence intervals (CI) and standard errors (SE). Table D. Summary statistics for sociodemographic characteristics and experiences of sexual violence among 35,349 females aged 13–24 years by drought exposure category. Table E. Definitions used in this study. Table F. Very dry districts with the top 10% of most intense dryness conditions considering the standardised dryness intensity. Table G. Convergence Assessment of MCMC Chains Across Drought Categories Using Rhat Values. Table H. Summary of Effective Sample Size (ESS) for MCMC Chains Across Drought Categories. Fig A. Dryness conditions in the Mwenezi district in Zimbabwe in a 48-month time window before data collection. Fig B. Dryness conditions in the Luwero district in Uganda in a 48-month time window before data collection. Fig C. Dryness conditions in the Karasburg district in Namibia in a 48-month time window before data collection. Fig D. Prior predictive checks plots for dryness conditions. Fig E. Posterior predictive checks plots for dryness conditions.
(DOCX)
