# Supplementary material for: Extreme drought and sexual violence against adolescent girls and young women: A multi-country population-based study
Source: PLOS Glob Public Health. 2025 Jun 26;5(6):e0004752. doi: 10.1371/journal.pgph.0004752 (PMC12200773; doi:10.1371/journal.pgph.0004752)
Supplement: S1 Text — Table A. Sexual violence measures used in the study, including questions for lifetime experience and follow-up questions to assess temporality and frequency. Table B. Questions included in the head of household questionnaire and measures used to estimate the wealth index. Table C. Weighted percentage prevalence of sexual violence victimisation in 14 countries using VACS data, including 95% confidence intervals (CI) and standard errors (SE). Table D. Summary statistics for sociodemographic characteristics and experiences of sexual violence among 35,349 females aged 13–24 years by drought exposure category. Table E. Definitions used in this study. Table F. Very dry districts with the top 10% of most intense dryness conditions considering the standardised dryness intensity. Table G. Convergence Assessment of MCMC Chains Across Drought Categories Using Rhat Values. Table H. Summary of Effective Sample Size (ESS) for MCMC Chains Across Drought Categories. Fig A. Dryness conditions in the Mwenezi district in Zimbabwe in a 48-month time window before data collection. Fig B. Dryness conditions in the Luwero district in Uganda in a 48-month time window before data collection. Fig C. Dryness conditions in the Karasburg district in Namibia in a 48-month time window before data collection. Fig D. Prior predictive checks plots for dryness conditions. Fig E. Posterior predictive checks plots for dryness conditions. (DOCX) [file pgph.0004752.s001.docx]

Supplementary material for

Extreme drought and sexual violence against adolescent girls and young women: a multi-country population-based study

Lucas Hertzog PhD*^1,2,3^, Marshall Makate PhD ^1^, David Chipanta PhD ^4^, Boladé Banougnin PhD ^5^, Martina Mchenga PhD ^6^, Gavin Pereira Prof ^1,2,7^, Sylvester Dodzi Nyadanu PhD ^1,3^, Jennifer Dunne PhD ^1^, Paula S. Tallman PhD ^8,9,10^, Shalean Collins PhD ^11^, Kefyalew Addis Alene PhD ^1,12,13^, Pauline Rousseau-Gueutin PhD ^14^, Astghik Mavisakalyan Prof ^15,16^, Ivan C. Hanigan PhD ^1,2,3,17^

^1^ Curtin School of Population Health, Faculty of Health Sciences, Curtin University, WA 6102, Australia.

^2^ WHO Collaborating Centre for Climate Change and Health Impact Assessment, WA 6102, Australia.

^3^ Healthy Environments and Lives (HEAL) National Research Network, Australia.

^4^ United Nations Joint Programme on HIV/AIDS (UNAIDS), Windhoek, Namibia.

^5^ United Nations Population Fund, West and Central Africa Region Office, Dakar, Senegal.

^6^ Centre for Social Science Research, University of Cape Town, Cape Town, South Africa.

^7^ enAble Institute, Faculty of Health Sciences, Curtin University, WA 6102, Australia.

^8^ Department of Anthropology, Loyola University Chicago, Chicago, Illinois, USA.

^9^ The Keller Science Action Center, The Field Museum of Natural History, Chicago, Illinois, USA.

^10^ Department of Social Welfare, Faculty of Social and Political Sciences, Padjadjaran University, Bandung, Indonesia.

^11^ Department of International Health & Sustainable Development, School of Public Health and Tropical Medicine, Tulane University, New Orleans, Louisiana, USA.

^12^ Geospatial and Tuberculosis Research Team, Telethon Kids Institute, Australia.

^13^ Department of Global Health and Social Medicine, Harvard Medical School, Boston, MA, USA.

^14^ Univ Rennes, Ecole des Hautes Etudes en Santé Publique, Rennes, France.

^15^ Bankwest Curtin Economics Centre, Curtin University, WA 6845, Australia.

^16^ ARC Centre of Excellence for the Elimination of Violence Against Women, Melbourne, Australia.

^17^ Centre for Safe Air, NHMRC CRE, Australia.

Correspondence:

*[lucas.hertzog@curtin.edu.au](mailto:lucas.hertzog@curtin.edu.au)

400.233, Curtin University, Kent St, Bentley WA 6102

Contents

[1. Sampling details 3](#_Toc197336106)

[2. Data preparation steps, outcome and wealth index description 5](#_Toc197336107)

[3. Detailed descriptive statistics of sexual violence victimisation 10](#_Toc197336108)

[4. Description of the drought measures 12](#_Toc197336109)

[5. Formal specification 21](#_Toc197336110)

[6. Missing data treatment 22](#_Toc197336111)

[7. Diagnostics 22](#_Toc197336112)

[7.1 Model specification 22](#_Toc197336113)

[Prior predictive check 22](#_Toc197336114)

[7.2 Details of the computation 24](#_Toc197336115)

[MCMC chain convergence 24](#_Toc197336116)

[MCMC chain resolution 25](#_Toc197336117)

[7.3 Posterior distribution 26](#_Toc197336118)

[References 27](#_Toc197336119)

# Sampling details

The surveys collected data from males and females aged 13-24, and separate surveys were conducted with the heads of households. For this analysis, only female interview responses were used, in addition to responses to the head of household questionnaire regardless of sex. Data access was not granted for Botswana (2016), Eswatini (2022), Jamaica (2022) and Rwanda (2015). Sampling documentation was not available or incomplete for Eswatini (2007), Honduras (2017), Lao PDR (2014), and Tanzania (2009), which hindered geo-coded data linkage. Haiti (2012) was excluded due to the sampling characteristics, which included internally displaced person (IDP) camps after an earthquake. Guatemala (2019) was excluded because it did not use a comparable method. The sampling frames for each country were based on the most recent national geopolitical subdivisions from available censuses used as master frames. A three-stage sample design was employed across all countries. In the first stage, primary sampling units (PSUs) were randomly selected using probability proportional to size from the master frame. A split sample approach was employed, with female and male PSUs in different communities, to safeguard the confidentiality of participants and reduce the likelihood of interviewing both a perpetrator and a victim from different sexes within the same community. More female PSUs were selected due to the implementation funder's interest in collecting more detailed data on females, a priority population with a disproportionately higher risk for violence. In the second stage, households within these PSUs were selected using equal probability systematic sampling within PSUs. In the third and final stage, one eligible individual aged 13-24 was randomly chosen from each selected household, depending on whether it was a male or female PSU, and administered the questionnaire when consent was granted for data collection.

Field pre-testing was conducted in each country before data collection started. Interviews utilised a structured questionnaire standardised across countries, with minor wording adaptations to accommodate local contexts. Responses were recorded on netbook computers.

Interviews with household heads were 15-minute assessments to collect socioeconomic information about the household. From February 2013 to July 2019, 35,349 females were interviewed across the selected 14 countries, and participants were interviewed in local languages. The overall response rate for females, defined as the percentage of the total number of completed survey responses out of the respective total number of survey participants, ranged between 47.3% (Moldova) and 96.2% (Lesotho). Individuals who were cognitively impaired or had significant physical disabilities, such as severe hearing or speech impairments, were excluded from participating in the survey. More details on the sampling approach can be found on the data custodian's website (<https://www.togetherforgirls.org/>).

# Data preparation steps, outcome and wealth index description

First, districts' names were renamed to match the exposure data exactly. Second, exposure, outcome (Table A) and control variables were recoded, and females were selected when the dataset included males. Third, a Principal Component Analysis (PCA) was conducted, including items used to estimate the wealth index (1) (see Table B). Variables were coded as binary categorical (0 or 1) and standardised using the *means* option, representing the proportion of observations with a value of 1. Variables are centred around this proportion in the standardisation process, and its standard deviation is used to scale it. By ensuring that every variable contributes to the PCA according to its relative variability within the dataset, this method makes it possible to present the generated components in a balanced manner (2). Based on the PCA's results, a fourth step was conducted to predict wealth index scores for each respondent. Fifth, following an approach used in the literature, a wealth quintile categorisation was performed, with the first and second quintiles considered less wealthy and the upper quintiles more wealthy (3,4). The sixth step involved merging all countries' data into a single dataset with country names as a variable. In R, the seventh step involved linking survey participants to exposure data using district names, declaring the survey design to account for the three-stage sampling, and ensuring all analyses yielded nationally representative results.

Table A Sexual violence measures used in the study, including questions for lifetime experience and follow up questions to assess temporality and frequency.

| **Measure** | **Question** | **Follow-up 1** | **Follow-up 2** |
| --- | --- | --- | --- |
| Touching without permission | Has anyone ever touched you in a sexual way without your permission, but did not try and force you to have sex? Touching in a sexual way without permission includes fondling, pinching, grabbing, or touching you on or around your sexual body parts. | How many times in your life has this happened? Did this happen to you within the past 12 months? | Now think about the last time ("last time" if response >1) this happened. Did this happen to you within the past 12 months? |
| Attempted forced sex | Has a boyfriend/romantic partner, girlfriend/romantic partner, ex-boyfriend/romantic partner, ex-girlfriend/romantic partner, husband, wife, ex-husband or ex-wife ever tried to make you have sex against your will but did not succeed? They might have tried to physically force you to have sex or they might have tried to pressure you to have sex through harassment or threats. | How many times in your life has anyone tried to make you have sex against your will but did not succeed? They might have tried to physically force you to have sex or they might have tried to pressure you to have sex through harassment or threats. | Now think about the last time ("last time" if response >1) this happened. Did this happen to you within the past 12 months? |
|  | Has anyone [else] ever tried to make you have sex against your will but did not succeed? If the individual did not have an intimate partner: They might have tried to physically force you to have sex or they might have tried to pressure you to have sex through harassment or threats. |  |  |
| Physically forced sex | Has a boyfriend/romantic partner, girlfriend/romantic partner, ex-boyfriend/romantic partner, ex-girlfriend/romantic partner, husband, wife, ex-husband or ex-wife ever physically forced you to have sex and did succeed? By physical force, we mean things like being pinned or held down or use of violence like pulling your hair, pushing, shoving, punching, using or threatening to use a weapon, or threatening to physically harm you or a loved one. We include experiences when you may or may not have fought back. | How many times in your life have you been physically forced to have sex? Remember, by physical force, we mean things like being pinned or held down or use of violence like pulling your hair, pushing, shoving, punching, using or threatening to use a weapon, or threatening to physically harm you or a loved one. We include experiences when you may or may not have fought back | Now think about the last time ("last time" if response >1) this happened. Did this happen to you within the past 12 months? |
|  | Has anyone [else] ever physically forced you to have sex against your will and did succeed? |  |  |
| Pressured into sex | Has a boyfriend/romantic partner, girlfriend/romantic partner, ex-boyfriend/romantic partner, ex-girlfriend/romantic partner, husband, wife, ex-husband or ex-wife ever pressured you in a non-physical way to have sex against your will and did succeed? Pressured means doing things like threatening you, harassing you, telling you lies, making promises about the future they knew were untrue, threatening to end your relationship, or threatening to spread rumors about you. | How many times in your life has someone pressured you in a non-physical way to have sex against your will and did succeed? Remember, pressured means doing things like threatening you, harassing you, telling you lies, making promises about the future they knew were untrue, threatening to end your relationship, or threatening to spread rumors about you. | Now think about the last time ("last time" if response >1) this happened. Did this happen to you within the past 12 months? |
|  | Has anyone [else] ever pressured you in a non-physical way to have sex against your will and did succeed? |  |  |

Table B Questions included in the head of household questionnaire and measures used to estimate the wealth index.

| **Component** | **Measure** | **Question** | **Options** |
| --- | --- | --- | --- |
| Sanitation | Source of drinking water | What is the main source of drinking water for members of your household? | Piper water |
|  |  |  | Tube well |
|  |  |  | Protected dug well |
|  |  |  | Unprotected dug well |
|  |  |  | Water from spring |
|  |  |  | Rainwater |
|  |  |  | Tanker water |
|  |  |  | Cart with small tank |
|  |  |  | Surface water (river/dam/lake/pond) |
|  |  |  | Stream/irrigation canal |
|  |  |  | Bottled water |
|  |  |  | Bagged water |
|  | Kind of toilet | What kind of toilet facility do members of your household mostly use? | Flush toilet |
|  |  |  | Pit latrine ventilated |
|  |  |  | Pit latrine with slab |
|  |  |  | Pit latrine without slab/open pit |
|  |  |  | Composting toilet |
|  |  |  | Bucket toilet |
|  |  |  | Hanging toilet/hanging latrine |
|  |  |  | No facility/bush/field |
|  |  |  | Other |
|  | Shared toiled | Do you share this facility with other households? | Yes/No |
| Assets | Assets (household) | Does your household have: | Electricity |
|  |  |  | A paraffin lamp |
|  |  |  | A gas lamp |
|  |  |  | A flashlight |
|  |  |  | Radio |
|  |  |  | Television |
|  |  |  | Mobile/cell telephone |
|  |  |  | Non-Mobile Telephone (landline) |
|  |  |  | Refrigerator |
|  |  |  | Freezer |
|  | Assets (household members) | Does any member of your household own: | Watch |
|  |  |  | Bicycle |
|  |  |  | Motorcycle or Scooter |
|  |  |  | Oxcart |
|  |  |  | Car or Truck |
|  |  |  | Cow |
|  |  |  | Goats/Sheep |
|  |  |  | Poultry (ex. Duck, chicken) |
|  |  |  | Dogs |
|  |  |  | Other animals (camels, horses, donkeys) |
|  | Land ownership | Does any member of this household own any agricultural land? | Yes/No |
|  | Farm animals | Does this household own any livestock, herds, other farm animals or poultry? | Yes/No |
|  | Banking | Does any member of this household have a bank account, including a Mobile Money account? | Yes/No |
| Dwelling | Cooking fuel | What type of fuel does your household mainly use for cooking? | Electricity |
|  |  |  | Liquefied petroleum gas (LPG) |
|  |  |  | Biogas |
|  |  |  | Kerosene |
|  |  |  | Coal, lignite |
|  |  |  | Charcoal |
|  |  |  | Wood |
|  |  |  | Straw/shrubs/grass |
|  |  |  | Animal dung |
|  |  |  | No food cooked in household |
|  |  |  | Other |
|  | Floor material | What is the main material of the dwelling floor? (observe or ask) | Earth/sand |
|  |  |  | Dung |
|  |  |  | Wood planks |
|  |  |  | Palm/bamboo |
|  |  |  | Broken bricks |
|  |  |  | Parquet/polished wood |
|  |  |  | Vinyl/asphalt strips |
|  |  |  | Ceramic tiles |
|  |  |  | Cement |
|  |  |  | Other |
|  | Roof material | What is the main material of the dwelling roof? (observe or ask) | No roof |
|  |  |  | Thatch/palm leaf |
|  |  |  | Rustic mat |
|  |  |  | Palm/bamboo/grass |
|  |  |  | Wood planks |
|  |  |  | Cardboard |
|  |  |  | Iron sheets |
|  |  |  | Wood |
|  |  |  | Calamine/cement fiber |
|  |  |  | Ceramic tiles |
|  |  |  | Cement |
|  |  |  | Roofing shingles |
|  |  |  | Other |
|  | Walls material | What is the main material of the dwelling walls? (observe or ask) | No walls |
|  |  |  | Cane/palm/trunks |
|  |  |  | Plastered dirt |
|  |  |  | Unplastered dirt |
|  |  |  | Bamboo/tree trunks with mud |
|  |  |  | Stone with mud |
|  |  |  | Plywood |
|  |  |  | Cardboard/tarp/tin/plastics |
|  |  |  | Construction waste |
|  |  |  | Reused wood |
|  |  |  | Cement |
|  |  |  | Stone with lime cement |
|  |  |  | Burnt bricks |
|  |  |  | Unburnt bricks |
|  |  |  | Cement blocks |
|  |  |  | Wood planks |
|  |  |  | Other |
|  | Household rooms | How many rooms are there in this household? | N of rooms |

# Detailed descriptive statistics of sexual violence victimisation

Table C Weighted percentage prevalence of sexual violence victimisation in 14 countries using VACS data, including 95% confidence intervals (CI) and standard errors (SE).

| Country | Lifetime sexual violence | CI- | CI+ | SE | Recent sexual violence | CI- | CI+ | SE |
| --- | --- | --- | --- | --- | --- | --- | --- | --- |
| Cambodia | 7.96 | 5.87 | 10.05 | 1.07 | 4.59 | 3.06 | 6.13 | 0.79 |
| Colombia | 21.16 | 14.05 | 28.26 | 3.63 | 14.99 | 9.5 | 20.47 | 2.8 |
| Côte d'Ivoire | 32.2 | 27.71 | 36.69 | 2.29 | 18.88 | 14.79 | 22.96 | 2.08 |
| El Salvador | 12.86 | 9.98 | 15.74 | 1.47 | 7.75 | 4.96 | 10.55 | 1.42 |
| Kenya | 25.21 | 21.64 | 28.77 | 1.82 | 12.61 | 10.01 | 15.21 | 1.32 |
| Lesotho | 19.04 | 17.24 | 20.84 | 0.92 | 11.05 | 9.93 | 12.17 | 0.57 |
| Malawi | 33.59 | 28.84 | 38.35 | 2.43 | 19.02 | 12.76 | 25.28 | 3.19 |
| Moldova | 20.19 | 16.75 | 23.63 | 1.76 | 12.6 | 9.54 | 15.65 | 1.56 |
| Mozambique | 21.24 | 18.42 | 24.06 | 1.44 | 12.01 | 9.46 | 14.57 | 1.3 |
| Namibia | 20.93 | 18.23 | 23.63 | 1.38 | 10.89 | 8.67 | 13.11 | 1.13 |
| Nigeria | 35.21 | 31.9 | 38.53 | 1.69 | 23.57 | 20.28 | 26.86 | 1.68 |
| Uganda | 43.98 | 40.52 | 47.43 | 1.76 | 23.79 | 20.69 | 26.88 | 1.58 |
| Zambia | 31.58 | 28.05 | 35.1 | 1.8 | 20.8 | 17.42 | 24.17 | 1.72 |
| Zimbabwe | 12.46 | 11.61 | 13.3 | 0.43 | 8 | 7.3 | 8.71 | 0.36 |

Table D Summary statistics for sociodemographic characteristics and experiences of sexual violence among 35,349 females aged 13–24 years by drought exposure category.

| Characteristic | Slight to Moderate, N = 25,867^1^ | Slight to Moderate & Recent and Long, N = 2,159^1^ | Prolonged and Extreme & Recent and Long, N = 1,853^1^ | Prolonged and Extreme & Very Dry & Recent and Long, N = 2,872^1^ | Very Dry & Recent and Long, N = 884^1^ | Very Dry, N = 1,674^1^ |
| --- | --- | --- | --- | --- | --- | --- |
| Age | 18.2 (3.5) | 18.1 (3.5) | 18.4 (3.4) | 17.9 (3.4) | 18.0 (3.4) | 17.8 (3.3) |
| Age group |  |  |  |  |  |  |
| 13-17 | 11,392 / 25,867 (44%) | 980 / 2,159 (45%) | 740 / 1,853 (40%) | 1,394 / 2,872 (49%) | 412 / 884 (47%) | 825 / 1,674 (49%) |
| 18-24 | 14,475 / 25,867 (56%) | 1,179 / 2,159 (55%) | 1,113 / 1,853 (60%) | 1,478 / 2,872 (51%) | 472 / 884 (53%) | 849 / 1,674 (51%) |
| Attending school | 13,061 / 25,867 (50%) | 895 / 2,159 (41%) | 600 / 1,853 (32%) | 1,468 / 2,872 (51%) | 482 / 884 (55%) | 986 / 1,674 (59%) |
| Ever married/relationship | 16,658 / 25,867 (64%) | 1,387 / 2,159 (64%) | 1,312 / 1,853 (71%) | 1,761 / 2,872 (61%) | 554 / 884 (63%) | 1,063 / 1,674 (64%) |
| Household is impoverished | 9,807 / 25,867 (38%) | 974 / 2,159 (45%) | 735 / 1,853 (40%) | 1,423 / 2,872 (50%) | 474 / 884 (54%) | 656 / 1,674 (39%) |
| Sexual violence (lifetime) | 4,923 / 25,867 (19%) | 666 / 2,159 (31%) | 525 / 1,853 (28%) | 654 / 2,872 (23%) | 163 / 884 (18%) | 309 / 1,674 (18%) |
| Sexual violence (previous 12 months) |  |  |  |  |  |  |
| No | 2,621 / 25,867 (10%) | 290 / 2,159 (13%) | 229 / 1,853 (12%) | 317 / 2,872 (11%) | 90 / 884 (10%) | 165 / 1,674 (9.9%) |
| Yes | 2,302 / 25,867 (8.9%) | 376 / 2,159 (17%) | 296 / 1,853 (16%) | 337 / 2,872 (12%) | 73 / 884 (8.3%) | 144 / 1,674 (8.6%) |
| Not Applicable | 20,944 / 25,867 (81%) | 1,493 / 2,159 (69%) | 1,328 / 1,853 (72%) | 2,218 / 2,872 (77%) | 721 / 884 (82%) | 1,365 / 1,674 (82%) |
| Country |  |  |  |  |  |  |
| Cambodia | 1,100 / 25,867 (4.3%) | 21 / 2,159 (1.0%) | 0 / 1,853 (0%) | 0 / 2,872 (0%) | 0 / 884 (0%) | 0 / 1,674 (0%) |
| Colombia | 937 / 25,867 (3.6%) | 195 / 2,159 (9.0%) | 77 / 1,853 (4.2%) | 115 / 2,872 (4.0%) | 42 / 884 (4.8%) | 0 / 1,674 (0%) |
| Côte d'Ivoire | 1,097 / 25,867 (4.2%) | 103 / 2,159 (4.8%) | 0 / 1,853 (0%) | 0 / 2,872 (0%) | 0 / 884 (0%) | 0 / 1,674 (0%) |
| El Salvador | 1,054 / 25,867 (4.1%) | 2 / 2,159 (<0.1%) | 0 / 1,853 (0%) | 0 / 2,872 (0%) | 0 / 884 (0%) | 0 / 1,674 (0%) |
| Kenya | 1,150 / 25,867 (4.4%) | 194 / 2,159 (9.0%) | 0 / 1,853 (0%) | 0 / 2,872 (0%) | 0 / 884 (0%) | 0 / 1,674 (0%) |
| Lesotho | 5,052 / 25,867 (20%) | 0 / 2,159 (0%) | 0 / 1,853 (0%) | 0 / 2,872 (0%) | 410 / 884 (46%) | 1,639 / 1,674 (98%) |
| Malawi | 1,029 / 25,867 (4.0%) | 0 / 2,159 (0%) | 0 / 1,853 (0%) | 0 / 2,872 (0%) | 0 / 884 (0%) | 0 / 1,674 (0%) |
| Moldova | 951 / 25,867 (3.7%) | 73 / 2,159 (3.4%) | 0 / 1,853 (0%) | 0 / 2,872 (0%) | 0 / 884 (0%) | 0 / 1,674 (0%) |
| Mozambique | 446 / 25,867 (1.7%) | 0 / 2,159 (0%) | 805 / 1,853 (43%) | 878 / 2,872 (31%) | 0 / 884 (0%) | 0 / 1,674 (0%) |
| Namibia | 3,139 / 25,867 (12%) | 18 / 2,159 (0.8%) | 0 / 1,853 (0%) | 817 / 2,872 (28%) | 219 / 884 (25%) | 18 / 1,674 (1.1%) |
| Nigeria | 1,396 / 25,867 (5.4%) | 93 / 2,159 (4.3%) | 0 / 1,853 (0%) | 168 / 2,872 (5.8%) | 92 / 884 (10%) | 17 / 1,674 (1.0%) |
| Uganda | 339 / 25,867 (1.3%) | 1,001 / 2,159 (46%) | 804 / 1,853 (43%) | 894 / 2,872 (31%) | 121 / 884 (14%) | 0 / 1,674 (0%) |
| Zambia | 891 / 25,867 (3.4%) | 0 / 2,159 (0%) | 0 / 1,853 (0%) | 0 / 2,872 (0%) | 0 / 884 (0%) | 0 / 1,674 (0%) |
| Zimbabwe | 7,286 / 25,867 (28%) | 459 / 2,159 (21%) | 167 / 1,853 (9.0%) | 0 / 2,872 (0%) | 0 / 884 (0%) | 0 / 1,674 (0%) |
| ^1^Mean (SD); n / N (%). | | | | | | |

# Description of the drought measures

Table D shows the definitions used in this study to construct the drought exposures. The SPEI may be calculated at different time scales which differ in the period over which water deficit/surplus is calculated: for example, 1-month periods (SPEI-1), 6-month periods (SPEI-6), 12-month periods (SPEI-12) etc. Shorter time scales are more responsive to acute drought and suit meteorological drought, while longer scales better represent hydrological drought. We used the SPEI-6 as the basis for our additional drought classifications. Their formulation supports the use of four distinct categories:

1. Slight to moderate (intensity), SPEI of less than zero to -1.
2. Very dry (intensity), top 10% of the months using the standard dryness intensity.
3. Recent and long (duration), count method >12 in the last 24 months.
4. Prolonged and extreme (duration and intensity), top 10% of months in drought that met the sum and count methods definition.

Table E Definitions used in this study.

| **Definitions** |  | **Description** |
| --- | --- | --- |
| Dry months |  | SPEI < -1. |
| Drought period |  | Starts after at least 5 consecutive dry months. |
| Count method |  | Counts how many months in drought after the 5^th^ month (e.g. 5 consecutive months, count method equals 1; 6 consecutive months, count method equals 2). |
| Sum method |  | Counts of how many months in drought in which the sum of the SPEI index is less or equal to -17.5. |
| Count and sum months |  | Total drought months that both criteria (count and sum) were met |
| Cycles of drought | Count method | Number cycles of droughts using the count method |
|  | Sum method | Number cycles of droughts using the sum method |
|  | Both methods |  |
| Standardised dryness intensity |  | Sum measure during periods below threshold (-17.5) divided by the average time per drought cycle (total number of drought months divided by the number of cycles in the sum method). |

We use three examples to demonstrate the drought exposures used in this study. First, the Mwenezi district in Zimbabwe. 80 participants in this study were interviewed, and exposure to dryness conditions is shown in Fig A:


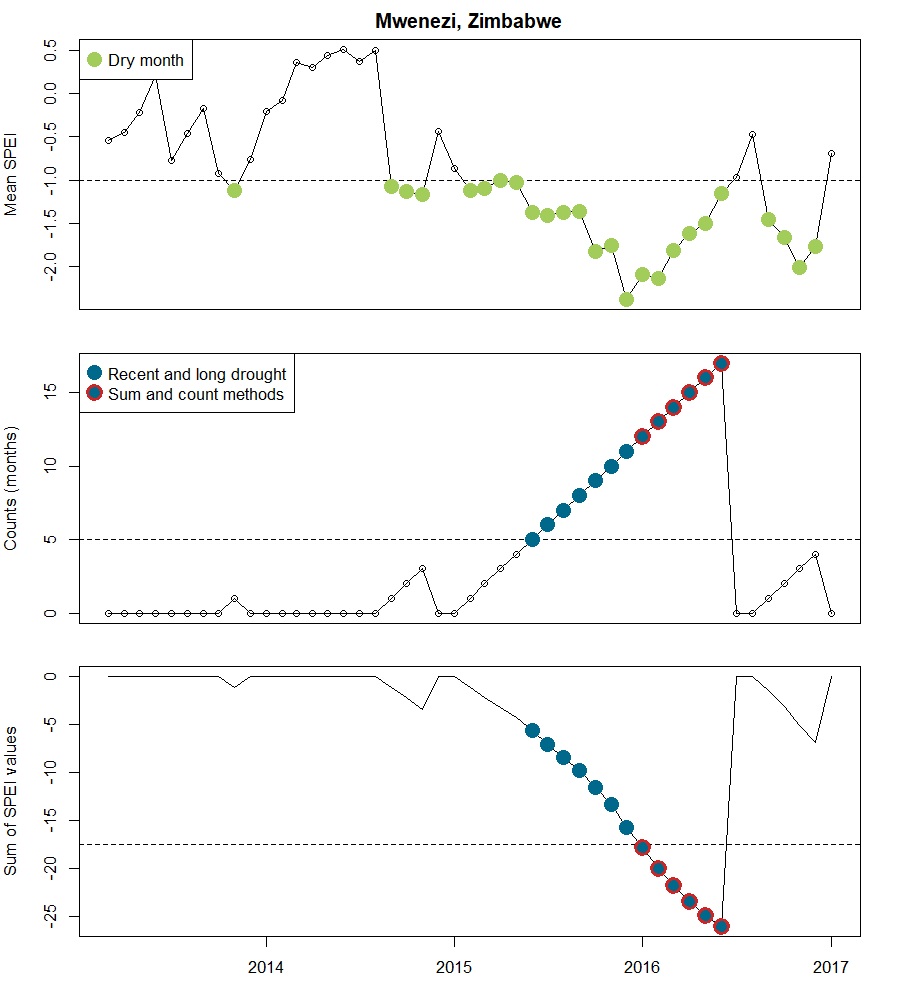


Fig A Dryness conditions in the Mwenezi district in Zimbabwe in a 48-month time window before data collection.

The top panel in the plot highlights, in green dots, months where SPEI values were less than -1, considered dry months (5). The middle panel shows the number of months in drought, with a threshold of 5 months, as demonstrated by the horizontal dotted line. In the selected period, Mwenzi had 13 months of drought after 2015. As demonstrated in the first panel, before that, there were dry months, but they were not considered 'in drought' as we use the 5-month threshold to consider the start of a drought. The 5-month threshold was based on the work from Australia which used observed drought declarations to find the optimum threshold to validate the rainfall-based drought indicators (5). In the first seven months of this drought period, the conditions of the sum method were not met (the threshold below -17.5 was also selected based on the Australian work comparing rainfall-based drought to drought declarations (5)), so they are flagged as 'recent and long drought' but not meeting both conditions. From the 8^th^ month of drought onwards, we see that the sum of SPEI values drops below the -17.5 threshold in the third panel (horizontal dotted line), and the blue dots with red borders are highlighted. This means that both conditions were met from the 8^th^ to the 13th month. Still, we do not consider this a 'prolonged and extreme' period. This is a category that estimates the top 10% of all cases in which both conditions were met (sum and count) across all districts in this study. In Mwenezi, the condition was met during six months, and it did not fall under the top 10% of most extreme cases.

In the second example, as demonstrated in Fig B, these conditions are met as we have 8 months considered in both methods (sum of months and count of SPEI values):


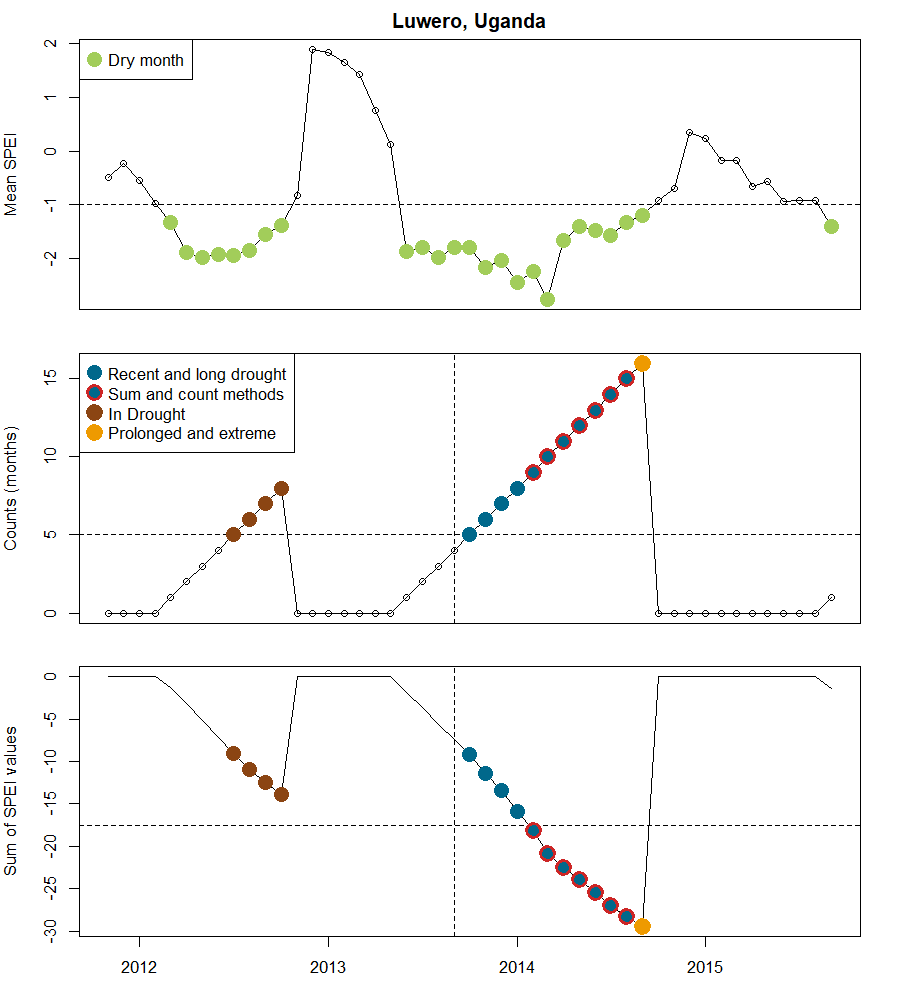


Fig B Dryness conditions in the Luwero district in Uganda in a 48-month time window before data collection.

In the district of Luwero in Uganda, 14 participants were interviewed for this study, and they are considered exposed to 'prolonged and extreme' drought. The sum and count methods are met for seven consecutive months, and 'prolonged and extreme' starts to be considered in the 8^th^. In Luwero, we also had a 4-month drought period before 2013, which is considered 'in drought' but does not impact recency-focused categories (such as recent and long) which is shown as the period on the righthand side of the vertical dotted line in panels 2 and 3, located at 24 months prior to interview. This can then be exemplified by our third example, the Karasburg district in Namibia, in which 14 participants were interviewed:


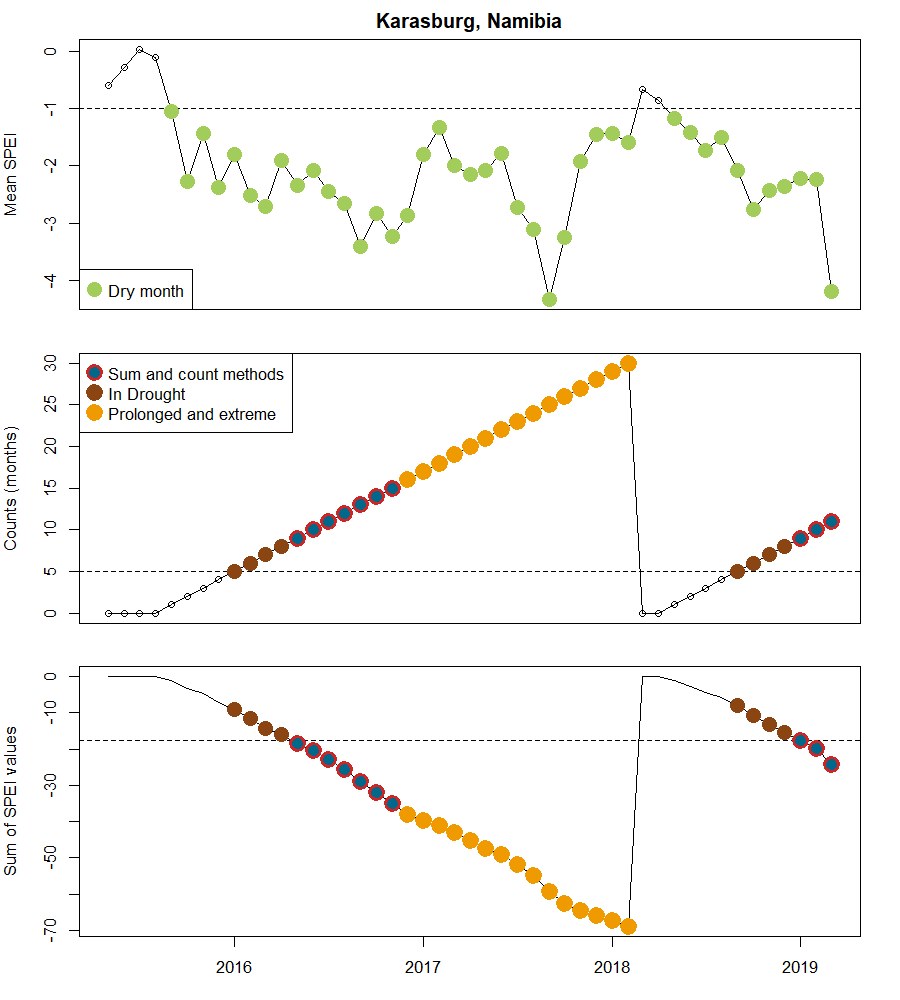


Fig C Dryness conditions in the Karasburg district in Namibia in a 48-month time window before data collection.

Karasburg is among the districts with worse drought conditions during our study period. As evidenced by the top panel, only six months had SPEI values higher than -1, which reflects the low precipitation levels found in Karasburg. In Fig C, we see that in the 8^th^ month, after simultaneous count and sum methods conditions, the threshold of the top 10% of worse districts in our study is met. The prolonged and extreme category is then applied, as the yellow dots demonstrate.

Finally, it is important to consider that participants may be exposed to multiple categories (see Fig 2 in the main manuscript). Karasburg, for example, scored -4.92, considering the standardised dryness intensity used to estimate the 'very dry' condition (intensity measure, see Table E). The 14 participants interviewed in Karasburg are classified as exposed to very dry, recent and long, and prolonged and extreme.

Table F Very dry districts with the top 10% of most intense dryness conditions considering the standardised dryness intensity.

| Country | Province* | District | Standardised dryness intensity |
| --- | --- | --- | --- |
| Colombia | Caquetá | Florencia | -2.018257 |
| Colombia | Amazonas | Leticia | -3.079056 |
| Colombia | Vaupés | Mitú | -3.366226 |
| Colombia | Amazonas | Puerto Nariño | -3.374606 |
| Colombia | Caquetá | Solano | -2.085164 |
| Colombia | Cauca | Timbiquí | -2.221259 |
| Colombia | Meta | Uribe | -2.205857 |
| Lesotho | Mokhotlong |  | -2.171041 |
| Lesotho | Mokhotlong |  | -2.171041 |
| Lesotho | Leribe |  | -2.002819 |
| Lesotho | Butha-Buthe |  | -2.401176 |
| Namibia | Oshikoto | Eengodi | -2.253028 |
| Namibia | Ohangwena | Eenhana | -5.949707 |
| Namibia | Ohangwena | Epembe | -5.949707 |
| Namibia | Oshikoto | Guinas | -2.168263 |
| Namibia | Kavango West | Kapako | -2.430748 |
| Namibia | Karas | Karasburg | -4.923518 |
| Namibia | Karas | Naminus (Luderitz) | -5.858312 |
| Namibia | Omusati | Okahao | -4.227440 |
| Namibia | Oshana | Okaku | -9.485960 |
| Namibia | Oshikoto | Okankolo | -2.487243 |
| Namibia | Oshana | Okatana | -9.480251 |
| Namibia | Oshikoto | Olukonda | -2.345964 |
| Namibia | Ohangwena | Omulonga | -6.153047 |
| Namibia | Oshikoto | Omuntele | -2.253433 |
| Namibia | Oshikoto | Onayena | -2.615050 |
| Namibia | Oshana | Ondangwa | -9.485960 |
| Namibia | Oshana | Ongwediva | -9.485960 |
| Namibia | Oshikoto | Oniipa | -5.956188 |
| Namibia | Oshikoto | Onyaanya | -2.281869 |
| Namibia | Ohangwena | Oshikango | -9.046597 |
| Namibia | Omusati | Oshikuku | -9.485960 |
| Namibia | Omusati | Outapi | -9.320234 |
| Namibia | Omusati | Tsandi | -8.972350 |
| Nigeria | Gombe | Akko | -3.050641 |
| Nigeria | Borno | Biu | -3.072503 |
| Nigeria | Kaduna | Chikun | -3.158538 |
| Nigeria | Gombe | Funakaye | -3.113421 |
| Nigeria | Adamawa | Guyuk | -3.434012 |
| Nigeria | Kaduna | Igabi | -3.139151 |
| Nigeria | Enugu | Igbo-Eze North | -2.694643 |
| Nigeria | Kaduna | Kaduna North | -3.153214 |
| Nigeria | Plateau | Kanam | -3.481620 |
| Nigeria | Kaduna | Kauru | -3.269735 |
| Nigeria | Kaduna | Kubau | -2.515046 |
| Nigeria | Gombe | Kwami | -3.126691 |
| Nigeria | Nasarawa | Lafia | -3.417904 |
| Nigeria | Plateau | Mangu | -4.095036 |
| Nigeria | Adamawa | Mubi North | -2.717065 |
| Nigeria | Adamawa | Numan | -3.356651 |
| Nigeria | Niger | Paikoro | -3.734435 |
| Nigeria | Plateau | Qua'an Pan | -3.486887 |
| Nigeria | Borno | Shani | -3.154840 |
| Nigeria | Kaduna | Soba | -2.477524 |
| Nigeria | Niger | Suleja | -3.826399 |
| Uganda | Northern | Yumbe | -3.338845 |
| Uganda | Northern | Arua | -3.445374 |
| Uganda | Northern | Gulu | -2.604267 |
| Uganda | Western | Kisoro | -2.256271 |
| Uganda | Western | Buhweju | -2.341843 |
| Uganda | Western | Rukungiri | -2.431561 |
| Uganda | Western | Isingiro | -2.081471 |
| Uganda | Western | Kabarole | -2.125587 |
| Uganda | Western | Bushenyi | -2.341331 |
| Uganda | Northern | Adjumani | -2.956277 |
| Uganda | Western | Kabale | -2.146395 |
| Uganda | Western | Ntungamo | -2.262308 |
| Uganda | Western | Mbarara | -2.341843 |
| Uganda | Western | Kamwenge | -2.125587 |
| Uganda | Western | Kanungu | -2.437510 |
| Uganda | Northern | Kole | -2.958348 |
| Uganda | Western | Kyegegwa | -1.999272 |
| Uganda | Northern | Maracha | -3.548564 |
| Uganda | Western | Kyenjojo | -2.125587 |
| Uganda | Northern | Nebbi | -3.028657 |
| Uganda | Western | Ntoroko | -2.017339 |
| Uganda | Northern | Nwoya | -3.038355 |
| Uganda | Western | Kiruhura | -2.092698 |
| Uganda | Northern | Zombo | -3.576562 |
| Uganda | Northern | Oyam | -2.958348 |
| Uganda | Western | Mitooma | -2.336391 |
| Uganda | Western | Sheema | -2.341843 |
| *Districts for Lesotho. | | | |

# Formal specification

The outcome variable in this model is the occurrence of sexual violence modelled as a Bernoulli distributed variable with probability $\pi_{i}$. The logit transformations of this probability are modelled as a linear combination of the predictor variables, including the drought exposures in separate equations (slight to moderate, very dry, recent and long, prolonged and extreme) and control variables (age, attending school, wealth, and relationship status). An intercept for each country is included to account for unobserved heterogeneity. The coefficients for the predictors ($\beta_{j}$) are assigned weakly informative normal priors due to the lack of previous studies to draw our assumptions, centred around zero with a standard deviation of 1. The random intercepts ($u_{\text{Country}\left( i \right)}$) are assumed to follow a normal distribution with mean zero and variance $\sigma^{2}$. Each of the following represents a separately estimated model examining the association between one type of drought exposure and sexual violence, controlling for the same set of covariates and including a country-level random intercept:

$${\text{Sexual}\text{ violence}}_{i}\sim\text{Bernoulli}\left( \pi_{i} \right)$$

Equation 1 (Slight to moderate):

$$\text{logit}\left( \pi_{i} \right)=\beta_{0}+\beta_{1}\text{Slight to moderate drought}_{i}+\gamma_{1}\text{Age}_{i}+\gamma_{2}\text{Attending school}_{i}+\gamma_{3}\text{Wealth}_{i}+\gamma_{4}\text{Relationship status}_{i}+u_{\text{Country}\left( i \right)}$$

Equation 2 (Very dry)

$$\text{logit}\left( \pi_{i} \right)=\beta_{0}+\beta_{1}\text{Very dry}_{i}+\gamma_{1}\text{Age}_{i}+\gamma_{2}\text{Attending school}_{i}+\gamma_{3}\text{Wealth}_{i}+\gamma_{4}\text{Relationship status}_{i}+u_{\text{Country}\left( i \right)}$$

Equation 3 (Recent and long)

$$\text{logit}\left( \pi_{i} \right)=\beta_{0}+\beta_{1}\text{Recent and long}_{i}+\gamma_{1}\text{Age}_{i}+\gamma_{2}\text{Attending school}_{i}+\gamma_{3}\text{Wealth}_{i}+\gamma_{4}\text{Relationship status}_{i}+u_{\text{Country}\left( i \right)}$$

Equation 4 (Prolonged and extreme)

$$\text{logit}\left( \pi_{i} \right)=\beta_{0}+\beta_{1}\text{Prolonged and extreme}_{i}+\gamma_{1}\text{Age}_{i}+\gamma_{2}\text{Attending school}_{i}+\gamma_{3}\text{Wealth}_{i}+\gamma_{4}\text{Relationship status}_{i}+u_{\text{Country}\left( i \right)}$$

$$\beta_{j}\sim\text{Normal}\left( 0,1 \right)$$

$$u_{\text{Country}\left( i \right)}\sim\text{Normal}\left( 0,\sigma^{2} \right)$$

This analysis followed the Bayesian Analysis Reporting Guidelines (BARG) (6), and full diagnostics are described in the following sections.

# Missing data treatment

Missing values frequency was low, ranging from 0.005% (age) to 0.45% (relationship status), and were addressed through imputation using the *missForest* package in R (7), executed over ten iterations and 100 trees, yielding a 0.135 out-of-bag (OOB) imputation error (variables imputed: age, school attendance, relationship status, sexual violence). Additional iterations and trees were tested but did not outperform the selected specifications. Exposure data for the island of San Andres in Colombia was unavailable, and 40 interviews were excluded.

# Diagnostics

## Model specification

### Prior predictive check

We conducted prior predictive checks to ensure that our chosen priors generate simulated data consistent with our assumed prior knowledge. Given the novelty of our study and the limited existing research on the relationship between drought on sexual violence, we used weakly informative priors (normal(0, 1)) for the model parameters.


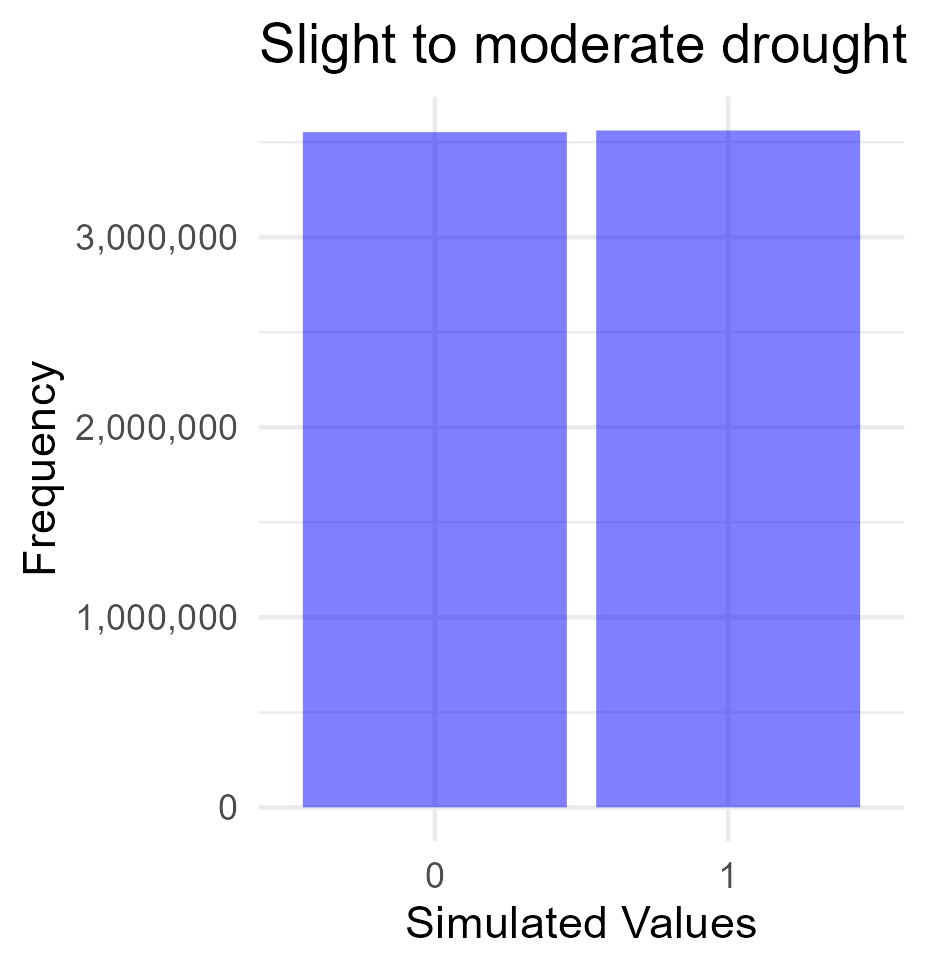

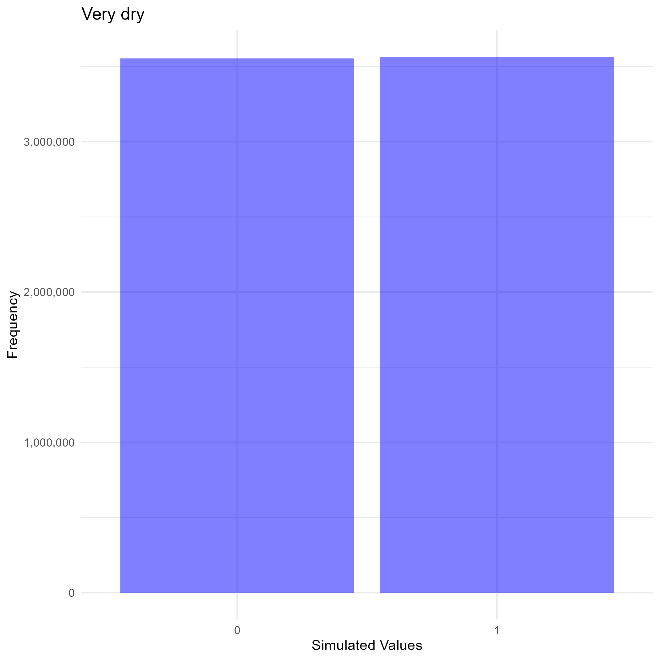

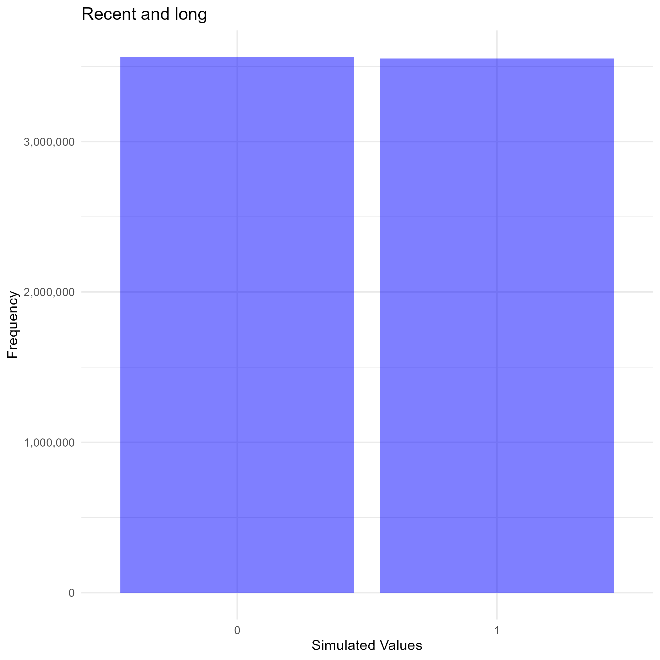

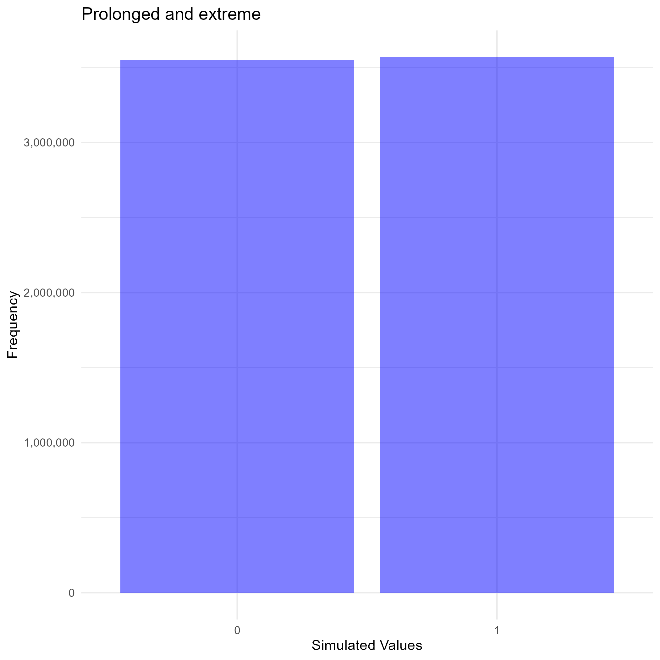


Fig D Prior predictive checks plots for dryness conditions.

The prior predictive checks show an even distribution of simulated outcomes between 0 and 1, indicating that our priors do not impose strong assumptions and allow the data to inform the model estimates effectively.

## Details of the computation

### MCMC chain convergence

Convergence of the Markov Chain Monte Carlo (MCMC) chains for each model using the Potential Scale Reduction Factor (Rhat) was assessed. The Rhat values for all parameters in each model were found to be close to 1, indicating good convergence. This suggests that the MCMC chains have mixed well, and the parameter estimates are reliable.

Table G Convergence Assessment of MCMC Chains Across Drought Categories Using Rhat Values.

| Parameter | Prolonged and extreme | Recent and long | Very dry | Slight to moderate |
| --- | --- | --- | --- | --- |
| (Intercept) | 1.007207 | 1.01186 | 1.012937 | 1.006861 |
| Sigma[adm0:(Intercept),(Intercept)] | 1.0069 | 1.005313 | 1.002293 | 1.006498 |
| age.imputed | 0.999542 | 0.999694 | 1.000473 | 1.000184 |
| b[(Intercept) adm0:Cambodia] | 1.007048 | 1.011999 | 1.012792 | 1.00713 |
| b[(Intercept) adm0:Colombia] | 1.007289 | 1.011971 | 1.012884 | 1.007063 |
| b[(Intercept) adm0:Cote_d'Ivoire] | 1.007132 | 1.011879 | 1.012844 | 1.006978 |
| b[(Intercept) adm0:El_Salvador] | 1.007018 | 1.011793 | 1.012736 | 1.007106 |
| b[(Intercept) adm0:Kenya] | 1.007232 | 1.012009 | 1.012838 | 1.007002 |
| b[(Intercept) adm0:Lesotho] | 1.007322 | 1.011825 | 1.012151 | 1.007363 |
| b[(Intercept) adm0:Malawi] | 1.007165 | 1.011869 | 1.012798 | 1.006978 |
| b[(Intercept) adm0:Moldova] | 1.007075 | 1.011755 | 1.012854 | 1.006765 |
| b[(Intercept) adm0:Mozambique] | 1.007191 | 1.011982 | 1.012738 | 1.006969 |
| b[(Intercept) adm0:Namibia] | 1.007182 | 1.011434 | 1.012665 | 1.007084 |
| b[(Intercept) adm0:Nigeria] | 1.007232 | 1.01192 | 1.01283 | 1.007059 |
| b[(Intercept) adm0:Uganda] | 1.007232 | 1.011886 | 1.012783 | 1.006975 |
| b[(Intercept) adm0:Zambia] | 1.00723 | 1.011864 | 1.012794 | 1.007068 |
| b[(Intercept) adm0:Zimbabwe] | 1.007064 | 1.011755 | 1.012758 | 1.007124 |
| b[(Intercept) adm0:_NEW_adm0] | 1.002022 | 1.000728 | 1.000746 | 1.00022 |
| Prolonged1 | 1.000191 | NA | NA | NA |
| schooll.imputed | 0.999661 | 0.99949 | 0.999705 | 0.999631 |
| log-posterior | 1.004485 | 1.003108 | 1.003273 | 1.011003 |
| relationship.imputed | 0.999377 | 1.000017 | 1.000498 | 1.000025 |
| mean_PPD | 1.000234 | 1.001167 | 0.999461 | 1.000237 |
| wealth | 1.00009 | 0.999302 | 1.001622 | 0.999482 |
| recent_long1 | NA | 1.000198 | NA | NA |
| very_dry1 | NA | NA | 1.000464 | NA |
| zero_to_moderate1 | NA | NA | NA | 1.001005 |

### MCMC chain resolution

Chains' resolution was assessed using the Effective Samle Size (ESS). Values were found to be high, indicating high-resolution chains. This suggests that the posterior estimates are based on a sufficient number of effective samples, providing confidence in the reliability of the results. The diagnostic table below provides a detailed summary of the ESS values for each parameter across all models:

Table H Summary of Effective Sample Size (ESS) for MCMC Chains Across Drought Categories.

| Parameter | Prolonged and extreme | Recent and long | Very dry | Slight to moderate drought |
| --- | --- | --- | --- | --- |
| (Intercept) | 358.9477 | 481.4555 | 375.4989 | 561.3626 |
| Sigma[adm0:(Intercept),(Intercept)] | 587.6304 | 696.4039 | 832.346 | 672.8502 |
| age.imputed | 4733.759 | 4463.4 | 4023.648 | 4084.014 |
| b[(Intercept) adm0:Cambodia] | 358.8638 | 478.5471 | 374.5321 | 559.7487 |
| b[(Intercept) adm0:Colombia] | 358.3119 | 476.6949 | 373.9134 | 558.1786 |
| b[(Intercept) adm0:Cote_d'Ivoire] | 359.0584 | 478.1484 | 373.8919 | 558.457 |
| b[(Intercept) adm0:El_Salvador] | 365.4001 | 478.896 | 375.4679 | 563.6302 |
| b[(Intercept) adm0:Kenya] | 359.6981 | 477.2582 | 373.5805 | 559.3433 |
| b[(Intercept) adm0:Lesotho] | 355.1995 | 481.1097 | 378.0539 | 562.7986 |
| b[(Intercept) adm0:Malawi] | 358.0707 | 477.9612 | 372.2586 | 560.0981 |
| b[(Intercept) adm0:Moldova] | 364.2114 | 481.3622 | 375.47 | 567.5577 |
| b[(Intercept) adm0:Mozambique] | 358.7907 | 478.546 | 374.5895 | 559.4854 |
| b[(Intercept) adm0:Namibia] | 366.0487 | 485.7649 | 382.166 | 565.1864 |
| b[(Intercept) adm0:Nigeria] | 358.3413 | 477.6768 | 374.595 | 558.6804 |
| b[(Intercept) adm0:Uganda] | 357.6438 | 478.1099 | 374.4579 | 559.794 |
| b[(Intercept) adm0:Zambia] | 359.9459 | 478.4764 | 375.236 | 559.02 |
| b[(Intercept) adm0:Zimbabwe] | 362.0757 | 479.7185 | 370.9429 | 560.7666 |
| b[(Intercept) adm0:_NEW_adm0] | 1882.485 | 2228.823 | 2265.988 | 1970.35 |
| Prolonged1 | 3743.31 | NA | NA | NA |
| schooll.imputed | 5103.772 | 4193.337 | 4719.79 | 4890.552 |
| log-posterior | 717.5162 | 746.3347 | 748.1058 | 670.1443 |
| relationship.imputed | 3806.767 | 3381.582 | 3954.975 | 4139.967 |
| mean_PPD | 4157.664 | 3859.716 | 4046.33 | 3626.77 |
| wealth | 4934.772 | 4552.775 | 4985.797 | 4832.439 |
| recent_long1 | NA | 3602.991 | NA | NA |
| very_dry1 | NA | NA | 3635.482 | NA |
| zero_to_moderate1 | NA | NA | NA | 4096.103 |

## Posterior distribution

The posterior predictive checks were performed to assess how well the Bayesian models mimic the observed data. Bar plots were generated for each exposure to compare the observed and predicted binary outcomes. The results indicate a close match between the observed and predicted values, suggesting that the models effectively capture the patterns in the data.


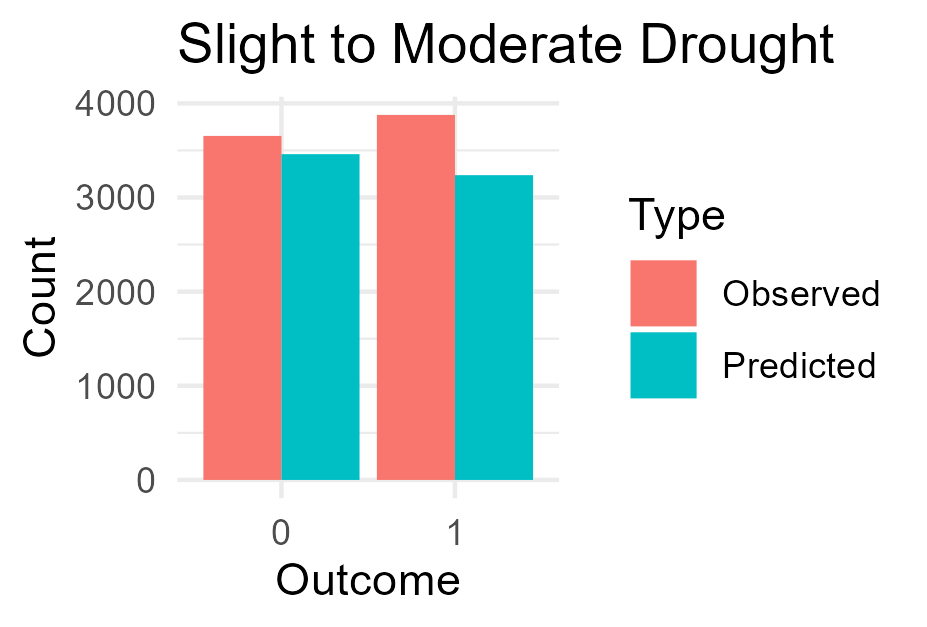

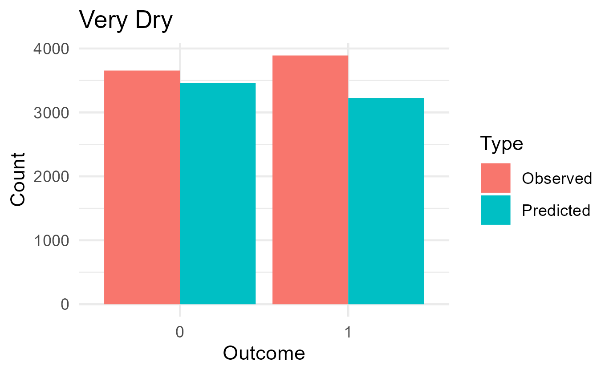

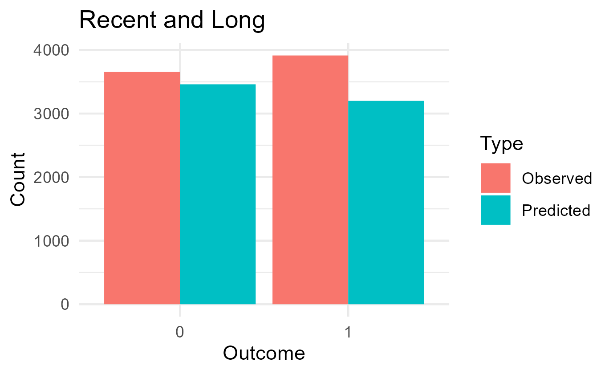

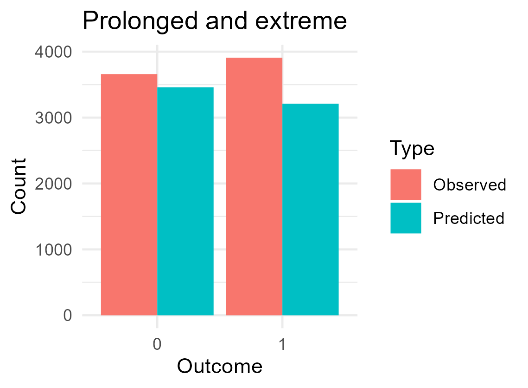


Fig E Posterior predictive checks plots for dryness conditions.

References

1. Rutstein S, Johnson K. The DHS Wealth Index. 2004 [cited 2023 Aug 11]; Available from: http://rgdoi.net/10.13140/2.1.2806.4809

2. Kolenikov S, Angeles G. Socioeconomic status measurement with discrete proxy variables: Is principal component analysis a reliable answer? Review of Income and Wealth. 2009 Mar;55(1):128–65.

3. Bamiwuye SO, Odimegwu C. Spousal violence in sub-Saharan Africa: does household poverty-wealth matter? Reprod Health. 2014 Dec;11(1):45.

4. Anyamele OD, Ukawuilulu JO, Akanegbu BN. The Role of Wealth and Mother’s Education in Infant and Child Mortality in 26 Sub-Saharan African Countries: Evidence from Pooled Demographic and Health Survey (DHS) Data 2003–2011 and African Development Indicators (ADI), 2012. Soc Indic Res. 2017 Feb;130(3):1125–46.

5. Hanigan IC, Butler CD, Kokic PN, Hutchinson MF. Suicide and drought in New South Wales, Australia, 1970–2007. Proc Natl Acad Sci USA. 2012 Aug 28;109(35):13950–5.

6. Kruschke JK. Bayesian Analysis Reporting Guidelines. Nat Hum Behav. 2021 Aug 16;5(10):1282–91.

7. Stekhoven DJ, Bühlmann P. MissForest—non-parametric missing value imputation for mixed-type data. Bioinformatics. 2012 Jan 1;28(1):112–8.
